# A Case for Pharmacogenomics in Management of Cardiac Arrhythmias

**DOI:** 10.1016/s0972-6292(16)30480-6

**Published:** 2012-04-30

**Authors:** Gaurav Kandoi, Anjali Nanda, Vinod Scaria, Sridhar Sivasubbu

**Affiliations:** 1 Department of Biotechnology, Delhi Technological University, Shahbad Daulatpur, Main Bawana Road, Delhi, India; 2 International Institute of Health Management Research, Plot no. 3, Sector 18A, Phase-II, Dwarka, New Delhi - 110075; 3 GN Ramachandran Knowledge Center for Genome Informatics, CSIR Institute of Genomics and Integrative Biology, Mall Road, Delhi 100 007, India; 4 Genomics and Molecular Medicine, CSIR Institute of Genomics and Integrative Biology, Mall Road, Delhi 100 007, India

**Keywords:** Arrhythmia, Pharmacogenomics, Personal genome, Genetic testing, Adverse drug reactions

## Abstract

Disorders of the cardiac rhythm are quite prevalent in clinical practice. Though the variability in drug response between individuals has been extensively studied, this information has not been widely used in clinical practice. Rapid advances in the field of pharmacogenomics have provided us with crucial insights on inter-individual genetic variability and its impact on drug metabolism and action. Technologies for faster and cheaper genetic testing and even personal genome sequencing would enable clinicians to optimize prescription based on the genetic makeup of the individual, which would open up new avenues in the area of personalized medicine. We have systematically looked at literature evidence on pharmacogenomics markers for anti-arrhythmic agents from the OpenPGx consortium collection and reason the applicability of genetics in the management of arrhythmia. We also discuss potential issues that need to be resolved before personalized pharmacogenomics becomes a reality in regular clinical practice.

Arrhythmias or disorders of the cardiac rhythm are not uncommon in clinical settings and one of the major causes of mortality and morbidity. Atrial Fibrillation is supposed to be rare in young healthy individuals unless without underlying cardiac pathology [10], while prevalent in the elderly and affects roughly around 2-5 Million individuals in the United States alone [2]. Ventricular fibrillation has a smaller incidence of close to 0.4 million [3]. Ventricular tachy-arrhythmias contribute significantly to the morbidity and mortality in patients with underlying coronary artery disease. It has been estimated that close to a half of deaths due to coronary artery disease is caused by ventricular arrhythmias [4]. Apart from the genetic and underlying cardiac disease as causes of cardiac rhythm abnormalities, a number of therapeutic agents, including drugs not directly used in the therapy of cardiac rhythm abnormalities have now been implicated to cause significant prolongation of QT interval and a form of ventricular arrhythmia, torsades de pointes, which is potentially fatal [5]. Recent reports also point to cardiac arrhythmias as one of the top causes for drug withdrawal and failure of clinical trials [6].

No major study on the incidence of arrhythmias or adverse drug reactions to anti-arrhythmic drugs throughout India has been performed. The lack of adequate epidemiological data in this important area has been highlighted in recent publications [4]. According to a report of arrhythmia care in India, published in 2002, the prevalence of patients with arrhythmias in the country is around 2 million [7]. Studies have also pointed to the high prevalence of asymptomatic arrhythmias in elderly patients [8]. According to the reports from the National pharmacovigilance programme, several cases of adverse drug reactions to anti-arrhythmic agents have been reported from people across India. Verapamil and Amiodarone have been reported to cause Steven Johnsons syndrome. Atenolol has been similarly reported to have adverse drug events like fatigue, cough and edema in a study conducted in South India [9]. Similar studies have shown Atenolol to be associated with around 4-5% of total adverse drug reactions reported.

Individuals vary widely in their response to therapeutic agents, and a large component of this variability is modulated through the genetic makeup of the individual. Apart from the variability in response, genetic variations are also now known to contribute significantly to Adverse Drug Reactions (ADRs). One of the earliest contributions to understanding of genomics of external agents have stemmed from the observations of the British physician Garrod, who proposed that defects in enzymatic pathways in unusual diseases of metabolism could produce unusual sensitivity to chemical agents. Molecular genetic dissection of congenital conditions in humans has contributed immensely to the overall understanding of the genetics of heart rhythm. The field has now grown by leaps and bounds with the advent of modern tools and techniques, which enables dissecting genetic phenomena at single base-pair resolution. The advent of genomics technologies has paved the way to deciphering the molecular genetic mechanisms of variability in response to therapeutic agents. This variability could be caused by genetic variations, which modulate the pharmacokinetics or pharmacodynamics of the drug. This could involve variations in genes involved in drug transport/metabolism right up to variations in drug-targets and off-targets. The field of understanding genetic variability in the response to drugs has now emerged into a full-fledged branch of biology - pharmacogenomics with the potential to significantly improve disease management. The field has also offered novel clues towards understanding mechanisms and pathways, which involves therapeutic agents.

The last couple of decades have seen enormous improvements in the management of cardiac arrhythmias. Due to limited benefits and safety related concerns, very few drugs have been successful and have been commonly used in the treatment of arrhythmias. The field has also seen the emergence of newer classes of drugs which function by normalizing the channel activity rather than blocking them. According to the popular Singh Vaughan Williams classification schema, drugs are placed based on the mechanism of action. The classification scheme has improved over time, presently including the miscellaneous class, which includes drugs which could not fit any of the previous classes. Recent years have seen a number of publications detailing the pharmacogenomics of anti-arrhythmic drugs [10-13]. Though many classes of anti-arrhythmic agents are not particularly used anymore currently in regular clinical practice except in special settings, the wealth of information on pharmacogenomics encompasses the commonly used classes of drugs as well. The Drugs and the genes involved in the pharmacogenomics of anti-arrhythmics and the respective references are summarized in Table 1[14-77].

Realizing the dream of personalized medicine is not without challenges and focused intervention. The major challenge in understanding the intricacies of genomic variations and deciphering the potential effects on pharmacokinetics and pharmacodynamics is the lack of comprehensive models of drug metabolism and action for many drugs. Understanding and charting drug pathways is the first step towards this dream. A systems level understanding of the drug pathways would enable us to overlay genomic variations and offer smart guesses on drugs that could be involved. The drug pathways for many drugs are complicated, involving multiple and sometimes redundant mechanisms for drug transport, metabolism and targets. Deciphering the pathways is the first step towards understanding how genetic variation could potentially contribute to the changes in functionality of critical components of the drug pathway. In addition, it also provides crucial insights into the molecular mechanisms of drug-drug and drug-environment interactions and how genetic variations could modulate this phenomenon. A comprehensive outline of anti-arrhythmic drugs and their drug pathways are summarized in Figure 1.

Modeling a disease process or pathway is the next critical step in understanding the molecular mechanisms and the genetic architecture of disease processes. Animal models such as rodents and mammals have been used successfully for modeling cardiac arrhythmias. Recently advances include the application of newer model systems for understanding pharmacogenomics principles. Model organisms like zebrafish, which are easy to maintain and study, have been shown to be useful in modeling pharmacological principles and potential mode of action of many therapeutic agents.

The major area that would require focused attention in the immediate future is towards standardized efforts to collate pharmacogenomics data and evidence to enable meta-analysis, while at the same time be able to keep pace with the latest avalanche of evidence brought to light by high throughput genomics studies including Genome-wide associations studies (GWAS). Community led approaches like PharmGKB (www.pharmgkb.org) and crowd-sourcing approaches like OpenPGx (www.openpgx.org) are the possible way forward, and both approaches should be organized complementary to each other. Apart from the data, the second focus area is computational tools and resources that can handle the high-throughput datasets. The availability of genome-wide scans as direct-to-consumer services has also provided an immense opportunity and challenge at the same time. With adequate computational tools and resources for interpretation of the data, this has the potential to lower the cost, while at the same time, widen the general acceptability of genetic testing. No healthcare intervention system is complete without adequate education and empowerment of the medical and paramedical professionals and the patients. For the success of widespread acceptability and application of pharmacogenomics testing for cardiac arrhythmias, appropriate focus and emphasis on awareness and healthcare education is essential.

These efforts should be complemented and supplemented by both systematic ways of collecting data and being able to analyze it to unravel emerging phenomena. This would necessitate creation of effective systems for systematic collection and sharing of clinical data, treatment protocols and outcome measures. This includes setting up of registries, which follow standard protocols, metadata and modes of data exchange. This also requires setting up collaborative and shared data resources and analytical approaches. In summary, seamless exchange of ideas, resources and knowhow between research laboratories and clinicians is essential to realize the dream of making pharmacogenomics based personalized medicine a reality.

## Figures and Tables

**Figure 1:**
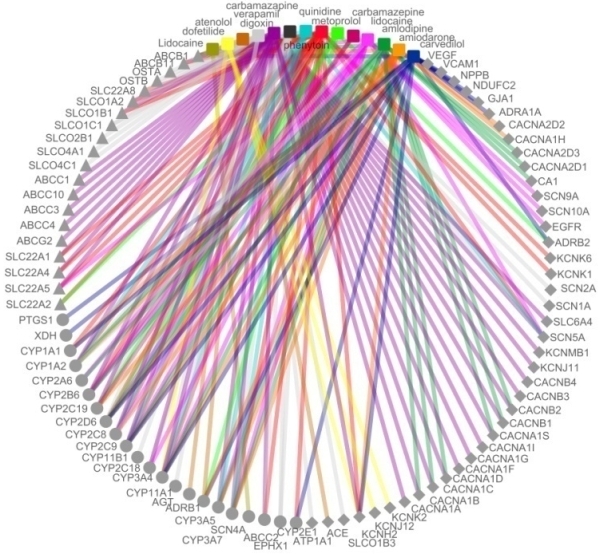
Overview of the drug pathways for anti-arrhythmic drugs. The coloured drugs and edges represent drugs while the metabolizing enzymes are marked as solid circles. The transporters and targets are represented as solid triangles and rhomboids respectively.

**Table 1:**
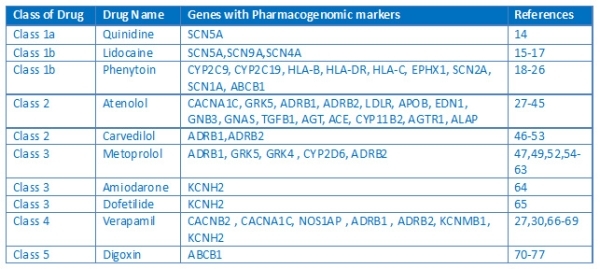
Summary of drugs, genes involved in the pharmacogenomics of anti-arrhythmic drugs.
